# Where is the ghost in the shell?

**DOI:** 10.1093/nc/niae015

**Published:** 2024-03-26

**Authors:** Veith Weilnhammer

**Affiliations:** Helen Wills Neuroscience Institute, University of California Berkeley, Berkeley, California 94720, United States

**Keywords:** consciousness, bistable perception, transcranial magnetic stimulation, parietal cortex

## Abstract

The neurobiology of conscious experience is one of the fundamental mysteries in science. New evidence suggests that transcranial magnetic stimulation of the parietal cortex does not modulate bistable perception. What does this mean for the neural correlates of consciousness, and how should we search for them?

The 1995 animated film *Ghost in the Shell* follows a cyborg named Major Motoko Kusanagi in a futuristic world, where technology is seamlessly integrated with human biology (Oshi 1995). Kusanagi ponders if her artificial brain generates true consciousness, questioning whether there is a *ghost* within her cybernetic *shell*. This provokes existential questions about the nature of consciousness: How do physical brains produce subjective experience? What are the essential features of biological or artificial systems that give rise to awareness? Can we upgrade consciousness?

Causal interventions on the artificial brains of the future may reveal how subjective experience emerges from the interacting components of a conscious machine. But progress can already be made in the biological brains of the present: in an elegant and rigorous study published in this issue of *Neuroscience of Consciousness*, Schauer and colleagues report that non-invasive brain stimulation of the parietal cortex does not modulate bistable perception. To understand their contribution in context, I will outline what bistable perception can teach us about conscious experience, and how causal interventions will bring us closer to identifying the neurocomputational correlates of consciousness.

During bistable perception, observers experience spontaneous transitions between two mutually exclusive interpretations of an ambiguous sensory input (Blake and Logothetis 2002). This happens, for example, when 2D stimuli cause us to perceive 3D objects (Fig. 1a), when static stimuli create apparent motion, or when conflicting images are presented to each eye (Blake and Logothetis 2002). In all these cases, conscious experience changes over time while the physical input remains constant. The phenomenon of bistable perception thus reveals that the correlates of consciousness go beyond a fixed mapping from sensory information to neural responses (Brascamp et al. 2018). This has sparked more than three decades of experiments on how the neural representation of the conscious or *dominant* interpretation differs from the unconscious or *suppressed* alternative, and what drives transitions between the two (Brascamp et al. 2018).

**Figure 1.**
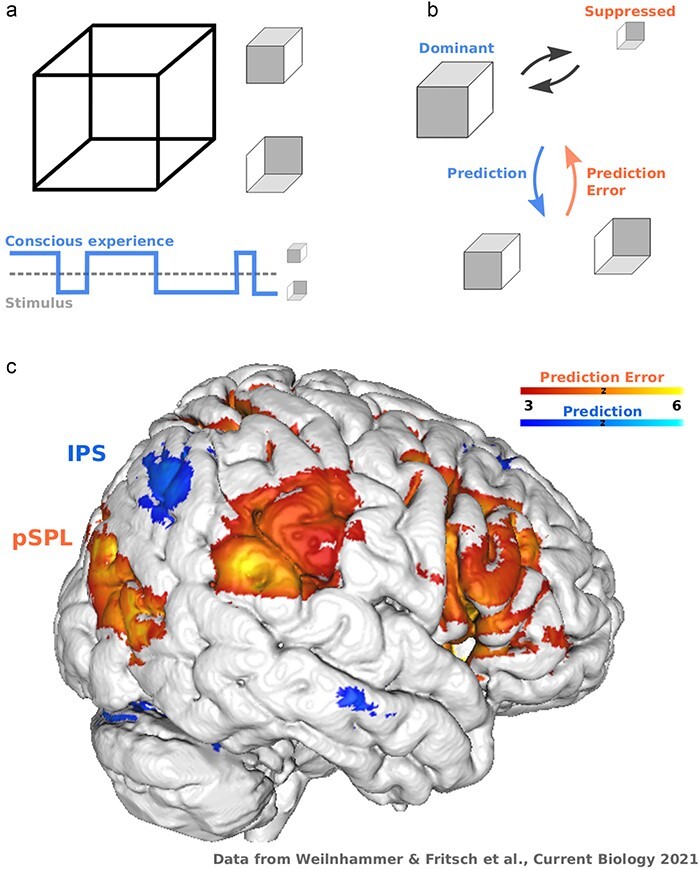
Concept. (a) Bistable perception happens when an ambiguous input is compatible with two mutually exclusive interpretations. In the example of the Necker cube, a 2D lattice (left) induces that experience of one of two cubes that differ with respect to spatial orientation (right). Bistable stimuli create a dissociation between the input (lower panel), which remains constant, and conscious experience, which changes over time. (b) Predictive processing posits that the dominant interpretation of a bistable stimulus (upper panel) reflects a prediction about the most likely cause of the physical input. Balanced stimulus evidence for the dominant and suppressed alternatives (lower panel) generates a prediction error that destabilizes perception, ultimately leading to a transition in conscious experience. (c) fMRI reveals a widespread representation of prediction errors during bistable perception (shown here for an individual participant from an experiment on the neural correlates of ambiguous structure-from-motion Weilnhammer et al. 2021), ranging from feature-selective regions in visual cortex to parietal and frontal cortex. From the perspective of model-based imaging, much less is known about the representation of the corresponding predictions (computed here as the negative prediction error response). Based on the behavioral effects of non-invasive brain stimulation, previous research has proposed that parietal cortex is divided into a posterior subregion in the SPL that encodes prediction errors, and an anterior subregion in the IPS that represents predictions during bistable perception. According to this model, inhibitory non-invasive brain stimulation of the SPL should therefore *reduce* the frequency of spontaneous transitions in conscious experience. In contrast, disrupting neural activity in the IPS should *increase* the frequency of spontaneous transitions in conscious experience

What we know so far is that sensory ambiguity elicits activity far beyond sensory cortices: while neurons in parietal and frontal cortex tend to follow the contents of conscious experience more closely, neural responses to the suppressed alternative can be found even at the latest stages of cortical processing (Kapoor et al. 2022). Compared to perceptual events induced by changes in visual stimulation, spontaneous transitions in conscious experience go along with increased brain activity in the frontoparietal network (Brascamp et al. 2018). Moreover, non-invasive brain stimulation of the frontoparietal network alters the dynamics of bistable perception (Brascamp et al. 2018), consistent with the hypothesis that regions in parietal and frontal cortex interact with sensory brain regions to gate access to consciousness (Weilnhammer et al. 2021, Dwarakanath et al. 2023).

Bistable perception is therefore not just an illusion that dissociates subjective experience from sensory information, but exposes a fundamental computational aspect of conscious experience. The stream of consciousness is *informative*: even though the data registered by our senses are inherently ambiguous, we are consciously aware of only one interpretation of the world, while competing alternatives are suppressed (Hohwy 2012). Conscious contents can thus be thought of as *predictions* about the sensory environment that are updated in response to *prediction errors* induced by conflicting information (Hohwy 2012) (Fig. 1b). This predictive processing view suggests a functional division within the neural correlates of consciousness, with predictive neurons that stabilize experience against random fluctuations in the input, and error neurons that support sensitivity to change. Their interaction is exposed during bistable perception, where neural responses to the suppressed alternative can be understood as prediction errors. Such prediction errors accumulate over time, destabilize the dominant interpretation of the input, and cause transitions in conscious experience (Hohwy et al. 2008, Weilnhammer et al. 2021).

Computational models are able to quantify dynamic prediction errors from the sequence of conscious experiences that participants report during bistable perception (Weilnhammer et al. 2017). These prediction errors successfully predict functional magnetic resonance imaging (fMRI) responses throughout the frontoparietal network (Weilnhammer et al. 2021) (Fig. 1c). Inhibitory transcranial magnetic stimulation (TMS) of the inferior frontal cortex, the most reliable neural correlate of prediction errors during bistable perception (Brascamp et al. 2018), reduces the frequency of spontaneous transitions in conscious experience (Weilnhammer et al. 2021). Distinct representations of predictions and prediction errors may also explain why TMS of parietal cortex has opposing effects on conscious experience (Brascamp et al. 2018), depending on whether neural activity is disrupted in a posterior subregion that represents destabilizing prediction errors (Kanai et al. 2010) (superior parietal lobe, pSPL), or in an anterior subregion that is associated with predictions that stabilize perception (Carmel et al. 2010, Kanai et al. 2011) (inferior parietal sulcus, IPS).

Despite these advances, the functional significance of the frontoparietal network for bistable perception is far from understood. Its causal role has been disputed by studies that associate parietal and frontal brain activity with introspection and reporting behavior—cognitive processes that occur not as a cause of, but in response to transitions in conscious experience (Brascamp et al. 2018). Moreover, non-invasive brain stimulation of the frontoparietal network has produced inconsistent results (Carmel et al. 2010, Kanai et al. 2010, 2011, Zaretskaya et al. 2010, de Graaf et al. 2011), which may be explained by limited sample size, variability in the response to TMS (Ozdemir et al. 2021), ongoing changes in dynamic brain state (Watanabe 2021), the bistable paradigm used, or differences in the protocol and target of TMS.

Schauer and colleagues address this issue in an impressive systematic investigation on how parietal continuous theta-burst stimulation (cTBS), an inhibitory TMS protocol (Huang et al. 2005), modulates bistable perception. They present data from three highly powered experiments in which they used (f)MRI-guided neuronavigation to ensure correct target localization, measured motor evoked potentials (MEPs) to assess cTBS response variability, controlled for unspecific effects via counter-balanced control stimulation at the vertex, and generalized across three bistable displays.

The data presented by Schauer and colleagues warrant the conclusion that ‘cTBS applied to parietal cortex is ineffective in modulating bistable perception’. In contradiction with the predictive processing hypothesis, Schauer and colleagues found no evidence for a functional division of parietal cortex into a posterior subregion in SPL that encodes destabilizing prediction errors (Kanai et al. 2010), and an anterior subregion in IPS that represents stabilizing predictions. In an elegant set of control analyses, the authors confirm that this null result cannot be explained by inter-individual differences in the susceptibility to cTBS, which they measured by comparing MEP amplitudes before and after cTBS to motor cortex. Moreover, while the frequency of transitions in conscious experience was correlated across three bistable displays, suggesting a common neural substrate, parietal cTBS effects were not. These results are important and surprising, since there is robust correlational evidence for a functional fractionation of parietal cortex (Megumi et al. 2015, Sandberg et al. 2016). Moreover, parietal cortex encodes prediction error signals that can also be found in inferior frontal cortex, where they predict the degree to which cTBS slows down bistable perception (Weilnhammer et al. 2021).

Given the large sample size reported by Schauer and colleagues, who did not find a significant cTBS effect even when pooling across experiments, it seems unlikely that interventions with even more power will reveal parietal subregions that have opposing causal roles during bistable perception. Instead, one avenue for future research may be to further increase the precision of non-invasive brain stimulation by computational modeling, which may help localize the neural correlates of predictions and predictions errors for fMRI-guided TMS in individual observers (Weilnhammer et al. 2021) (Fig. 1c). Another promising direction may be to test whether the impact of parietal cortex on bistable perception depends on dynamic brain states in these regions (Watanabe 2021, Weilnhammer et al. 2023). Finally, causal experiments based on alternative non-invasive stimulation protocols (Zaretskaya et al. 2010), invasive brain stimulation during neurosurgery or in non-human primates, as well as case–control studies in patients with structural lesions in the frontoparietal network (e.g. after ischemic stroke) may further disambiguate the role of frontoparietal cortex in bistable perception.

The impressive contribution by Schauer and colleagues indicates that the makers of Ghost in the Shell, which is set in the year 2029, were optimistic with respect to how quickly a mechanistic understanding of subjective experience would develop. At this time, it seems that we are still far away from dissecting the brain into individual subregions with distinct computational functions for consciousness. Systematic investigations like the study by Schauer and colleagues are therefore vital to inform our search for the neural correlates of conscious experience, and will pave the way toward non-invasive brain stimulation as an individualized treatment for people who suffer from altered states of consciousness such as hallucinations.

## Data Availability

The data underlying this article will be shared on reasonable request to the corresponding author.

## References

[R1] Blake R, Logothetis NK. Visual competition. *Nat Rev Neurosci* 2002;3:13–21. 11823801 10.1038/nrn701

[R2] Brascamp J, Sterzer P, Blake R et al. Multistable perception and the role of the frontoparietal cortex in perceptual inference. *Annu Rev Psychol* 2018;69:77–103. 28854000 10.1146/annurev-psych-010417-085944

[R3] Carmel D, Walsh V, Lavie N et al. Right parietal TMS shortens dominance durations in binocular rivalry. *Curr Biol* 2010;20:R799–800. 20869603 10.1016/j.cub.2010.07.036

[R4] de Graaf TA, de Jong MC, Goebel R et al. On the functional relevance of frontal cortex for passive and voluntarily controlled bistable vision. *Cereb Cortex* 2011;21:2322–31. 21385836 10.1093/cercor/bhr015

[R5] Dwarakanath A, Kapoor V, Werner J et al. Bistability of prefrontal states gates access to consciousness. *Neuron* 2023;111:1666–83.e4. 36921603 10.1016/j.neuron.2023.02.027

[R6] Hohwy J . Attention and conscious perception in the hypothesis testing brain. *Front Psychol* 2012;3:96. 10.3389/fpsyg.2012.00096 PMC3317264 22485102

[R7] Hohwy J, Roepstorff A, Friston K et al. Predictive coding explains binocular rivalry: an epistemological review. *Cognition* 2008;108:687–701. 18649876 10.1016/j.cognition.2008.05.010

[R8] Huang YZ, Edwards MJ, Rounis E et al. Theta burst stimulation of the human motor cortex. *Neuron* 2005;45:201–6. 15664172 10.1016/j.neuron.2004.12.033

[R9] Kanai R, Bahrami B, Rees G et al. Human parietal cortex structure predicts individual differences in perceptual rivalry. *Curr Biol* 2010;20:1626–30. 20727757 10.1016/j.cub.2010.07.027 PMC2949566

[R10] Kanai R, Carmel D, Bahrami B et al. Structural and functional fractionation of right superior parietal cortex in bistable perception. *Curr Biol* 2011;21:R106–107. 21300270 10.1016/j.cub.2010.12.009 PMC3084447

[R11] Kapoor V, Dwarakanath A, Safavi S et al. Decoding internally generated transitions of conscious contents in the prefrontal cortex without subjective reports. *Nat Commun* 2022;13:1535. 10.1038/s41467-022-28897-2 PMC8940963 35318323

[R12] Megumi F, Bahrami B, Kanai R et al. Brain activity dynamics in human parietal regions during spontaneous switches in bistable perception. *NeuroImage* 2015;107:190–7. 25512040 10.1016/j.neuroimage.2014.12.018 PMC4306523

[R13] Oshii M . Ghost in the shell. *Based on “Ghost in the Shell” by Masamune Shirow* 1995, Manga Entertainment.

[R14] Ozdemir RA, Boucher P, Fried PJ et al. Reproducibility of cortical response modulation induced by intermittent and continuous theta-burst stimulation of the human motor cortex. *Brain Stimul* 2021;14:949–64. 34126233 10.1016/j.brs.2021.05.013 PMC8565400

[R15] Sandberg K, Blicher JU, Del Pin SH et al. Improved estimates for the role of grey matter volume and GABA in bistable perception. *Cortex* 2016;83:292–305. 27639213 10.1016/j.cortex.2016.08.006 PMC5282065

[R16] Watanabe T . Causal roles of prefrontal cortex during spontaneous perceptual switching are determined by brain state dynamics. *eLife* 2021;10:e69079. 10.7554/eLife.69079 PMC8631941 34713803

[R17] Weilnhammer V, Fritsch M, Chikermane M et al. An active role of inferior frontal cortex in conscious experience. *Curr Biol* 2021;31:2868–80.e8. 33989530 10.1016/j.cub.2021.04.043

[R18] Weilnhammer V, Stuke H, Hesselmann G et al. A predictive coding account of bistable perception - a model-based fMRI study. *PLoS Comput Biol* 2017;13:e1005536. 10.1371/journal.pcbi.1005536 PMC5448813 28505152

[R19] Weilnhammer V, Stuke H, Standvoss K et al. Sensory processing in humans and mice fluctuates between external and internal modes. *PLoS Biol* 2023;21:e3002410. 10.1371/journal.pbio.3002410 PMC10732408 38064502

[R20] Zaretskaya N, Thielscher A, Logothetis NK et al. Disrupting parietal function prolongs dominance durations in binocular rivalry. *Curr Biol* 2010;20:2106–11. 21093263 10.1016/j.cub.2010.10.046

